# Linkage disequilibrium and effective population size when generations overlap

**DOI:** 10.1111/j.1752-4571.2012.00289.x

**Published:** 2012-08-08

**Authors:** John D Robinson, Gregory R Moyer

**Affiliations:** Conservation Genetics Lab, Fish Technology Center, U.S, Fish and Wildlife ServiceWarm Springs, GA, USA

**Keywords:** age structure, computer simulations, effective number of breeders, LDNe, microsatellites, *N*_*b*_, *N*_*e*_, SPIP

## Abstract

Estimates of effective population size are critical for species of conservation concern. Genetic datasets can be used to provide robust estimates of this important parameter. However, the methods used to obtain these estimates assume that generations are discrete. We used simulated data to assess the influences of overlapping generations on the estimates of effective size provided by the linkage disequilibrium (LD) method. Our simulations focus on two factors: the degree of reproductive skew exhibited by the focal species and the generation time, without considering sample size or the level of polymorphism at marker loci. In situations where a majority of reproduction is achieved by a small fraction of the population, the effective number of breeders can be much smaller than the per-generation effective population size. The LD in samples of newborns can provide estimates of the former size, while our results indicate that the latter size is best estimated using random samples of reproductively mature adults. Using samples of adults, the downwards bias was less than approximately 15% across our simulated life histories. As noted in previous assessments, precision of the estimate depends on the magnitude of effective size itself, with greater precision achieved for small populations.

## Introduction

The effective population size (*N*_*e*_) is the size of an idealized population that experiences the same magnitude of genetic drift and exhibits the same rate of inbreeding as the biological population under consideration (Wright 1931). This parameter is important in conservation, as it can help to explain contemporary patterns of genetic variation in natural populations and by definition describes the rate of inbreeding accumulation and loss of genetic variation. Inbreeding depression and, to a lesser extent, the loss of genetic diversity in turn influence the extinction risk of wild populations (Frankham 2005). For this reason, knowledge of *N*_*e*_ is crucial for species of conservation concern. Life-history data can be used to estimate *N*_*e*_ (Waples et al. 2011), but a more common approach is to use genetic data for this purpose. These estimates can be obtained from four primary genetic signals: heterozygote excess, linkage disequilibrium (LD), temporal variance in allele frequencies, and the amount of standing genetic diversity present in natural populations (see Wang 2005 for a review). The different means of estimating *N*_*e*_ apply to different windows of time in a population's demographic history (Waples 2005) and to different definitions of *N*_*e*_ (e.g., variance effective size, inbreeding effective size, and eigenvalue effective size; see Crandall et al. 1999). These factors can make values calculated from different approaches difficult to compare even if they are produced from data sampled from the same population.

Two of the most commonly used genetic estimates of *N*_*e*_ are those provided by the temporal variance in allele frequencies (Waples 1989) and levels of LD in the population (Laurie-Ahlberg and Weir 1979). These two methods both provide information on contemporary effective size, but differ in their required datasets and in the time frames to which they apply. For the temporal method, at least two samples are required, preferably separated by multiple generations, while the LD method provides estimates based on a single sample. Estimates from the LD method should primarily reflect the inbreeding effective size of the preceding generation (Hare et al. 2011), although population expansions or contractions can lead to transient biases in *N*_*e*_ that persist for a handful of generations (Waples 2005). In contrast, the temporal method estimates the harmonic mean *N*_*e*_ over the period separating the samples (Waples 2005). Both methods perform best when *N*_*e*_ is small, as the signal (drift in allele frequencies or LD) is largest in these cases (Waples and Do 2010).

Additionally, both methods deal primarily with discrete generation models, an assumption that is violated in natural populations where generations often overlap. Previous researchers have derived formulas for *N*_*e*_ in species with overlapping generations (Hill 1972, 1979) and investigated the influence of age structure on the ratio of effective to census population size (Nunney 1991, 1993). Waples and Yokota (2007) sought to determine the utility of the temporal method for estimating the per-generation *N*_*e*_ in species with overlapping generations. They found that the bias introduced by overlapping generations could be substantial unless samples were spaced apart by several generations. When this is not possible, other options include sampling consecutive cohorts (Jorde and Ryman 1995) and weighting allele frequencies by the reproductive value of individual age classes (Waples and Yokota 2007). Another option, when the age and sex of individuals are known, is to use the estimator by parentage assignments (EPA), proposed by Wang et al. (2010). In practice, authors often choose to ignore the potential bias introduced by the life history of the focal species (Waples 2010). Palstra and Ruzzante (2008) provide further confirmation of the importance of considering these factors; in their review, *N*_*e*_ estimates were substantially higher when discrete generation models were uncritically applied to species with overlapping generations, indicative of a downwards bias in these estimates (Palstra and Ruzzante 2008).

To date, the influence of overlapping generations on estimates from the LD method has not been assessed (Waples 2010). Waples and Do (2010) hypothesized that estimates from the LD method should approach per-generation *N*_*e*_ in species with overlapping generations, given an appropriate sampling regime (where the number of consecutive cohorts sampled is close to the generation time). In this study, we sought to address this hypothesis using simulated datasets. We considered the effects of life-history variation (focusing on increases in reproductive variability across age classes and generation time) and sampling methodology on *N*_*e*_ estimation. By assessing the bias introduced to estimates of *N*_*e*_ by the use of the LD method in species with overlapping generations, our results can inform sampling designs in future studies that seek to estimate one of the most important population parameters in evolutionary and conservation biology. As in any simulation study, the range of life-history scenarios considered limits the generality of our results.

## Materials and methods

We designed four life-history scenarios [based on the survival and reproduction of sparrows as given in Table 2 of Waples and Yokota (2007)] to assess the influences of age structure on the estimates of *N*_*e*_ from the LD method. These scenarios shared age-specific survival probabilities, total numbers of offspring produced per year (*N*_1_), and maximum life spans, but differed in the relative reproductive contribution of age classes (Table 1). Reproduction in scenarios A and B was constant or nearly so across age classes, whereas in scenario C, fecundity increased with age. In scenario D, an extreme case of reproductive skew was modeled; the fecundity of the oldest age class was far higher than that during the rest of the life span. These scenarios allowed us to assess the performance of the LD method when estimating both the effective number of breeders per year (*N*_*b*_) and the per-generation *N*_*e*_. These two values were similar for scenarios with nearly constant age-specific fecundity (e.g., A and B), but diverged increasingly as reproduction became skewed to the older age groups. All four scenarios were constrained to exhibit stable population sizes by setting the number of newborns produced at *N*_1_ = 1000 individuals.

**Table 1:** Demographic parameters for the four primary simulated life histories

Age (*x*)	Shared parameters		Scen. A	Scen. B*	Scen. C	Scen. D
				
	*s*_*x*_	*l*_*x*_	*b*_*x*_	*b*_*x*_	*b*_*x*_	*b*_*x*_
1	0.180	1	0	0	0	0
2	0.528	0.180	2.724	2.546	1.426	1.732
3	0.537	0.095	2.724	2.754	2.852	1.732
4	0.529	0.051	2.724	2.921	4.278	1.732
5	0.519	0.027	2.724	3.130	5.704	1.732
6	0	0.014	2.724	3.339	7.130	27.717
		*N*_1_	1000	1000	1000	1000
		*T*_*G*_†	2.910	3.000	3.569	4.036
		*N*_*e*_†	378.8	370.3	302.7	213.8
		*N*_*b*_†	367	365	273	85

Scenarios share age-specific survival probabilities (*s*_*x*_) and, therefore, survivorship to age probabilities (*l*_*x*_), but differ in their age-specific fecundities (*b*_*x*_). The numbers of newborns (*N*_1_), generation lengths (*T*_*G*_), and effective sizes per generation (*N*_*e*_) and per year (*N*_*b*_) are given.

* Sparrow example from Table 2 of Waples and Yokota (2007).

† Values calculated using agene (Waples et al. 2011).

The four life histories in Table 1 all model relatively short-lived species. To determine whether a much longer generation time would influence estimates provided by the LD method, we simulated data for an additional three scenarios with 20 age classes each (Table 2). Two of these scenarios were based on life-history data for Atlantic sturgeon (*Acipenser oxyrhynchus*), while the third was simulated to match the life history of the fat threeridge mussel (*Amblema neislerii*). For sturgeon, we modeled one population (large *N*_*e*_) that produced 8000 newborns each year and another (small *N*_*e*_) producing 450 newborns per year. For the mussel, only a small *N*_*e*_ scenario was modeled (*N*_1_ = 106 newborns). Life-history parameters were derived from previous publications for both the sturgeon (Moyer et al. 2012) and mussel (Miller 2008) examples. The software package agene (Waples et al. 2011) was used to calculate expected values for *N*_*e*_ and *N*_*b*_ for all modeled scenarios. The effective sizes of the populations we simulated were small to focus our study on a region of parameter space where the LD method performs well (Waples and Do 2010), but were somewhat larger than the average estimated *N*_*e*_ across studies considered in a recent review of estimates provided by the temporal method (Palstra and Ruzzante 2008).

**Table 2:** Demographic parameters for sturgeon and mussel life-history scenarios

Age (*x*)	Sturgeon*			Mussel†		
	
	*s*_*x*_	*l*_*x*_	*b*_*x*_	*s*_*x*_	*l*_*x*_	*b*_*x*_
1	0.753	1	0	0.850	1	0
2	0.763	0.753	0	0.913	0.850	0
3	0.776	0.574	0	0.913	0.776	0
4	0.788	0.446	0	0.913	0.708	0.035
5	0.793	0.351	0	0.913	0.646	0.035
6	0.803	0.278	0.007	0.913	0.589	0.035
7	0.811	0.223	0.022	0.913	0.538	0.035
8	0.816	0.181	0.085	0.913	0.491	0.035
9	0.826	0.148	0.209	0.913	0.448	0.089
10	0.826	0.122	0.932	0.913	0.409	0.089
11	0.830	0.101	1.016	0.913	0.373	0.089
12	0.833	0.084	1.286	0.913	0.340	0.089
13	0.841	0.070	1.651	0.913	0.310	0.089
14	0.840	0.059	2.203	0.913	0.283	0.089
15	0.837	0.049	2.347	0.913	0.258	0.089
16	0.842	0.041	2.462	0.913	0.236	0.296
17	0.833	0.035	2.270	0.913	0.215	0.296
18	0.844	0.029	2.727	0.913	0.196	0.296
19	0.844	0.024	2.768	0.913	0.179	0.296
20	0	0.021	2.828	0	0.164	0.296
*N*_1_	8000/450				106						
*T*_*G*_‡	14.150				13.621						
*N*_*e*_‡	6210.3/349.3				499.7						
*N*_*b*_‡	3691/207				360						

Age-specific survival probabilities (*s*_*x*_), survivorship to age probabilities (*l*_*x*_), and age-specific fecundities (*b*_*x*_) are given, along with numbers of newborns (*N*_1_), generation lengths (*T*_*G*_), and effective sizes per generation (*N*_*e*_) and per year (*N*_*b*_). For sturgeon simulations, *N*_1_, *N*_*e*_, and *N*_*b*_ are given for both simulated scenarios (large *N*_*e*_/small *N*_*e*_).

* Life history of sturgeon based on Moyer et al. (2012).

† Life history of mussels based on Miller (2008).

‡ Values calculated using agene (Waples et al. 2011).

Microsatellite datasets for simulated populations under each of these scenarios were generated using SPIP (Anderson and Dunham 2005). This software allows the simulation of pedigrees and genetic datasets, in this case multilocus microsatellite genotype data. All simulations were initiated with population numbers close to the stable age distributions that result from the survival probabilities given in Tables 1 and 2. This practice should minimize the influence of fluctuations in population size early in the simulations, which might lead to bias in our estimates (*n.b*. bias in the LD method arising from population fluctuations lasts only a handful of generations; Waples 2005).

Both SPIP (Anderson and Dunham 2005) and agene (Waples et al. 2011) assume stable population size; age-specific fecundities (*b*_*x*_; Tables 1 and 2) in both were specified as relative values and rescaled for analyses and simulations. Random mating was assumed, and sex ratios were 1:1 for all but the sturgeon life history, where a male-skewed sex ratio was simulated (74% male; Moyer et al. 2012). We used options in SPIP to specify a constant cohort size (*N*_1_) and the ‘discard all’ option early in the simulation. Simulations were run for a total of 100 generations, after which genotypes at twenty unlinked loci were obtained for all individuals. Each locus was initiated with nine alleles, but this serves as a maximum allelic richness for the population as SPIP does not model mutation, but allows the loss of alleles through genetic drift in small populations (Anderson and Dunham 2005). Additionally, our SPIP simulations assumed that all newborns survive to age 1 (*s*_0_ = 1.0) and that no reproduction occurred until age 2 or later. One thousand replicate simulations were conducted under each of the scenarios outlined earlier.

The resulting genotype files were subsampled using scripts written for the R statistical computing environment (R Development Core Team 2012). Our sampling strategies included a newborn-only sample (to estimate *N*_*b*_), a sample with equal representation from each of the age classes (e.g., 20 individuals sampled from each of five age classes), a random mixed-age sample, and random samples of only reproductively mature adults. Additionally, for two of the life-history scenarios (A and C), we sampled increasingly nonrandom portions of the population (the three youngest age classes, the two youngest age classes, and the two oldest age classes). Similarly, for the sturgeon (small *N*_*e*_ only) and mussel scenarios, we also simulated samples from the five youngest age classes, the ten youngest age classes, the fifteen youngest age classes, and ten nonconsecutive age classes. All samples consisted of one hundred diploid individuals, except for samples drawn from the two oldest age classes for scenarios A and C, in which fewer than one hundred individuals were available.

Estimates of effective size (both *N*_*e*_ and *N*_*b*_) were calculated using the software package LDne (Waples and Do 2008). Low-frequency alleles can bias estimates of *N*_*e*_ provided by the LD method (Waples and Do 2010). For this reason, LDne (Waples and Do 2008) includes options to exclude alleles at frequencies lower than a specified criterion (*P*_crit_). We report estimates at three allele exclusion criteria (*P*_crit_ = 0.05, 0.02, and 0.01), but given the size of our simulated datasets (*n* = 100 individuals), we based our inferences on estimates provided using *P*_crit_ = 0.02 (Waples and Do 2010). We used the harmonic mean to measure the accuracy of the estimates (Waples and Do 2010). Harmonic means were calculated across all replicate simulations, including those that produced negative estimates of *N*_*e*_. The inclusion of negative estimates had little overall effect on these values, as they were exceedingly rare (19 negative values from a total of 114 000 estimates represented by data, given in the Appendices A and B). We used the coefficient of variation (CV), calculated from positive estimates only, to assess precision. The CV of our *N*_*e*_ estimates was compared with the value expected, given the average number of independent allelic comparisons for each group of simulations [E(CV) calculated using eqn 3 from Waples and Do (2010)]. It is important to note that Waples and Do (2010) recognized that eqn 3 underestimated the CV, because of upward skew in effective size estimates. We also report the 2.5% and 97.5% quantiles of the distribution of estimated values as an additional measure of precision.

## Results

**Figure 1:**
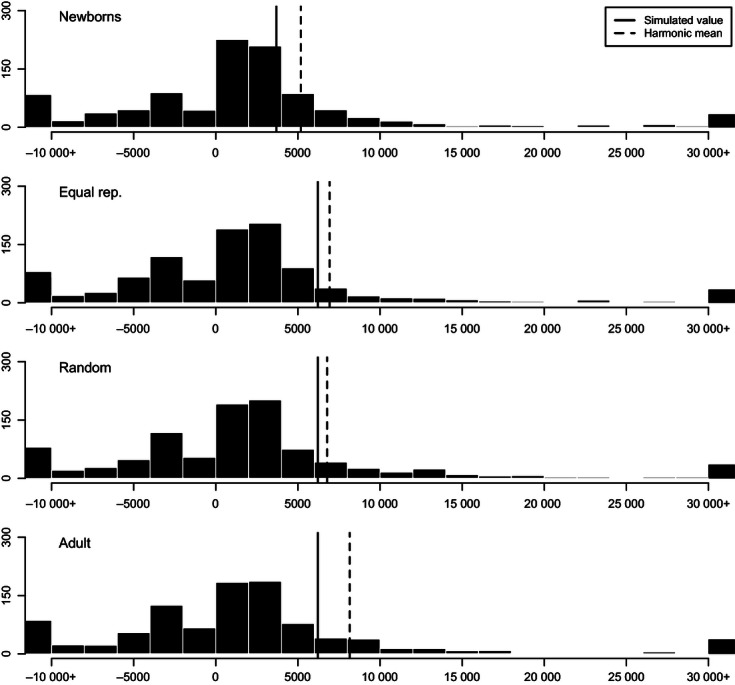
Distribution of *N*_*b*_ (1st panel) and *N*_*e*_ estimates (panels 2–4) for large *N*_*e*_ sturgeon (*N*_*e*_ = 6210.3) simulations. Each panel corresponds to estimates from a single-sampling strategy. Samples of the youngest age class (newborns) were used to estimate *N*_*b*_, while all other sampling strategies should estimate *N*_*e*_. Samples were drawn with equal representation of all age classes (equal rep.), randomly from the entire population (including newborns – random), or randomly from reproductively mature adults. Harmonic mean estimated effective size (dotted line) and simulated (parametric) values (solid line) are shown for each group.

Based on life-history data for our four primary scenarios, generation length (*T*_*G*_) ranged from 2.91 to 4.035, *N*_*e*_ ranged from 213.8 to 378.8, and *N*_*b*_ ranged from 85 to 367 (Table 1). Generation length was much longer for sturgeon and mussel life histories (14.15 and 13.621), and effective sizes per generation were 6210.3/349.3 (large *N*_*e*_/small *N*_*e*_) and 499.7, respectively. In these same scenarios, *N*_*b*_ values were substantially lower than per-generation effective sizes, at 3691/207 (large *N*_*e*_/small *N*_*e*_) for sturgeon and 360 for mussels. The performance of the LDne estimator depended on the underlying life history, the sampling strategy, and the magnitude of *N*_*e*_. In large *N*_*e*_ sturgeon simulations, estimates lacked precision and were frequently negative (Fig. 1), implying very large *N*_*e*_ (Waples and Do 2010). For this reason, we focused our analyses on the other scenarios, where *N*_*e*_ was simulated at values <500. Estimates from the three allele exclusion criteria employed in LDne were similar (Appendices A and B); thus, we used a *P*_crit_ value of 0.02 for all subsequent figures.

**Figure 2:**
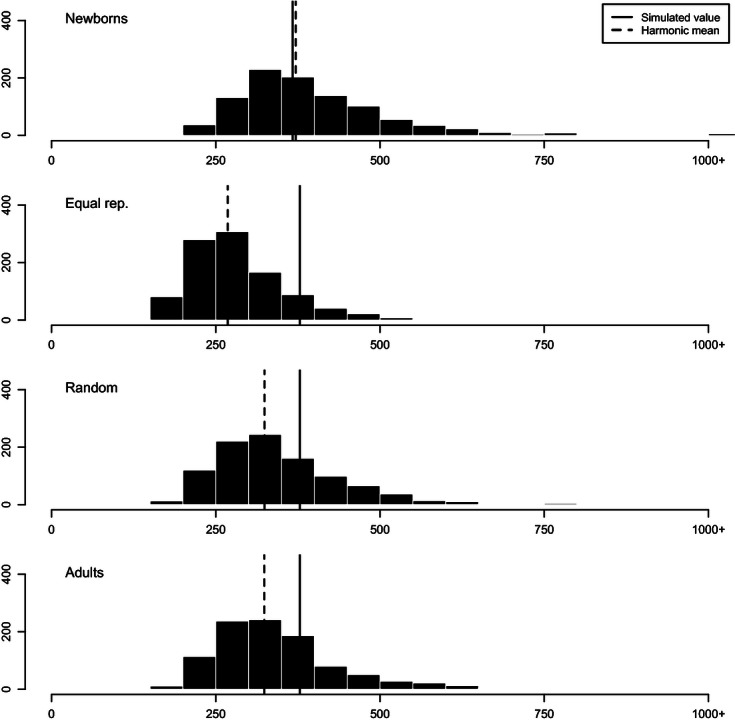
Distribution of *N*_*b*_ (1st panel) and *N*_*e*_ estimates (panels 2–4) for scenario A simulations. Under this life history, fecundity is constant across age classes. Each panel corresponds to estimates from a single-sampling strategy (strategies and point values follow Fig. 1).

In general, estimates of *N*_*e*_ were biased downwards, while estimates of *N*_*b*_ were biased upwards across a large region of parameter space (Appendices A and B). Additionally, estimates of *N*_*b*_ appeared to be highly influenced by the underlying life history. Scenarios A–C showed very little bias in estimates of *N*_*b*_ (<5%), while simulations for sturgeon, mussels, and scenario D life histories had bias ranging from 14% to 36% (Appendices A and B). In scenarios A, B, and C (Fig. 2, S1 and 3), patterns across sampling designs were highly consistent with one another. Newborn samples provided accurate estimates of *N*_*b*_, while estimates of *N*_*e*_ were most accurate when random samples of all age classes, or of only reproductively mature individuals, were considered. The patterns in scenario D simulations were substantially different from those in scenarios A–C; newborn samples yielded estimates of *N*_*b*_ that were biased upwards by approximately 36% (Appendix A), and the most accurate estimates of per-generation *N*_*e*_ were given by samples that included equal numbers of individuals in each age class (Fig. S2). The large relative bias in *N*_*b*_ estimates in scenario D simulations was not solely due to the smaller true *N*_*b*_ in these simulations; absolute bias in this case was approximately threefold greater than in estimates from scenarios A, B, and C. Results from sturgeon (Fig. 4) and mussel (Fig. S3) simulations matched those of scenarios A–C more closely, although estimates of *N*_*b*_ showed an upwards bias similar to that seen in scenario D. Additionally, all simulated scenarios where reproductive effort was skewed to later age classes (C, D, sturgeon, mussel) showed a consistent pattern for smaller estimated *N*_*e*_ when newborns were included in random mixed-age samples (Appendices A and B). Across our simulated life histories and sampling strategies, bias was less than approximately 15% for mixed-age samples of reproductively mature adults.

**Figure 3:**
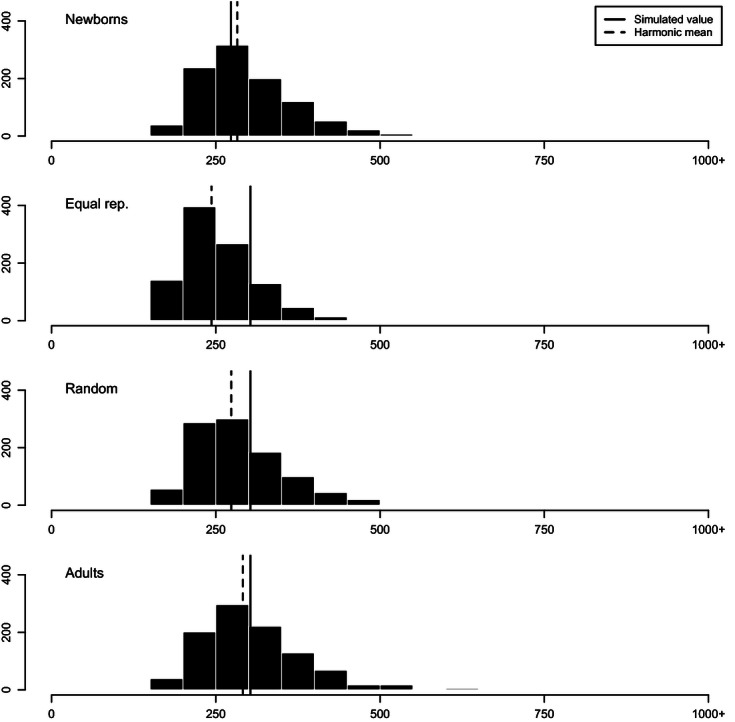
Distribution of *N*_*b*_ (1st panel) and *N*_*e*_ estimates (panels 2–4) for scenario C simulations. For this life history, fecundity increases linearly with age. Each panel corresponds to estimates from a single-sampling strategy (strategies and point values follow Fig. 1).

**Figure 4:**
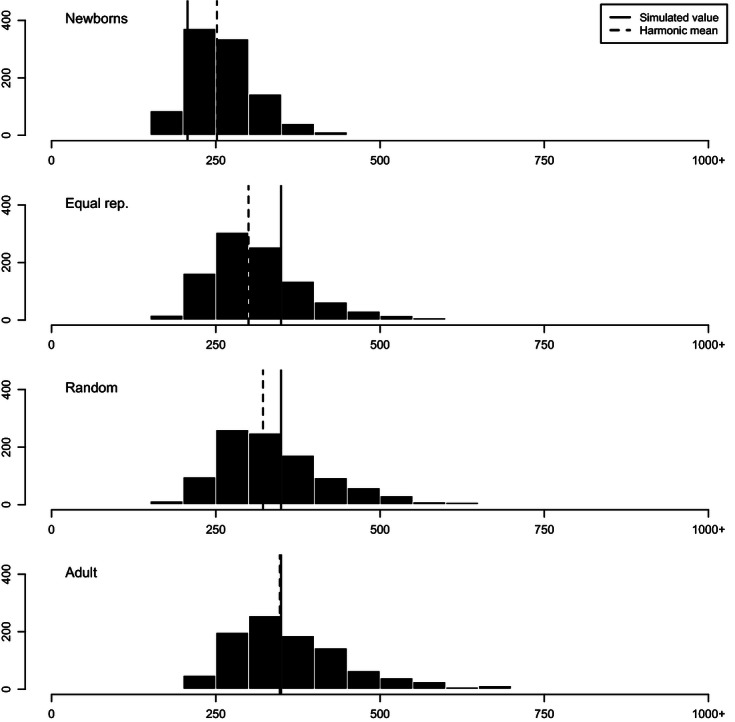
Distribution of *N*_*b*_ (1st panel) and *N*_*e*_ estimates (panels 2–4) for small *N*_*e*_ sturgeon (*N*_*e*_ = 349.3) simulations. Each panel corresponds to estimates from a single-sampling strategy (strategies and point values follow Fig. 1).

**Figure 5:**
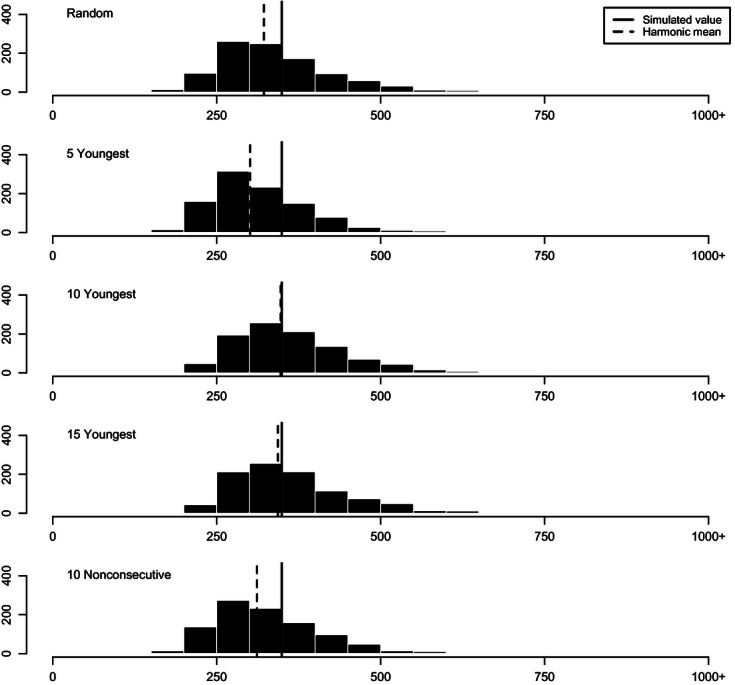
Distribution of *N*_*e*_ estimates for decreasingly random samples from small *N*_*e*_ sturgeon (*N*_*e*_ = 349.3) simulations. Each panel corresponds to estimates from a single-sampling strategy. The number of age classes included in the sample varied among strategies. We include random samples of all age classes for comparison with results in Fig. 4 (point values depicted follow Fig. 1).

To further assess the influence of sampling design on *N*_*e*_ estimates, we examined cases where the entire population could not be sampled. In these cases, the bias in our estimates changed direction for simulations under scenarios A and C (Figs S4 and S5; Appendix A). For sturgeon (Fig. 5) and mussel (Fig. S6) simulations, samples of consecutive age classes gave estimates with greater accuracy (bias <5%) than those including nonconsecutive age classes (bias 10–20%). With the exception of the mussel life history, greater accuracy was attained when the samples included a large number of age classes (Appendices A and B).

We used the CV to assess precision of our estimates of effective size. This measure may not be perfectly suited for this use, particularly when the distribution of estimates is skewed upwards (Waples and Do 2010). For this reason, in Appendices A and B, we report the observed CV, the expected CV from eqn 3 of Waples and Do (2010), and the 2.5% and 97.5% quantiles of the distribution of estimated effective sizes for each simulated sampling scheme x life history combination. Despite the inflation of the CV because of upwards skew, coefficients of variation in our estimates were typically between 0.2 and 0.4 (Appendices A and B). Several exceptions to this trend were apparent; newborn samples from scenario A and samples of the two oldest age classes in this scenario both had CV > 0.9 (Appendix A). In these cases, the lack of precision was because of extreme outliers; for the newborn samples, six datasets (from 1000 replicates) produced estimates of *N*_*b*_ > 1000 (Fig. 2). When only the two oldest age classes were considered, 61 simulated datasets estimated *N*_*e*_ > 1000. An additional six datasets (not included in the CV or shown in Fig. S4) gave negative estimates (down to *N*_*e*_ = −84 000). Extreme positive values inflated CV for these sampling strategies. For samples that did not include all age classes, precision increased with the number of classes sampled in scenarios A and C (Figs S4 and S5), but not for sturgeon (Fig. 5) and mussel (Fig. S6) life histories. Estimates provided by subsamples of the mussel age classes were also relatively imprecise when compared with other life histories (Fig. S6). This was most likely due to the larger *N*_*e*_ in these simulations. From our simulations, and as previously noted (Waples and Do 2010), it was apparent that precision decreased as effective size increased (compare Figs 1 and 4).

When precision was assessed using the 2.5% and 97.5% quantiles for estimates provided by *P*_crit_ = 0.02, it was apparent that (across our simulated life histories and sampling schemes) 95% of the estimated values for a combination were generally distributed in a range with a width between 70% and 140% of the true (simulated) effective size (Appendices A and B). For instance, 95% of the estimated *N*_*e*_ values for adult samples under life-history scenario C were between 195.5 and 519.1 (simulated *N*_*e*_ = 302.7). Wider ranges of estimates were found when only subsets of the population were sampled (e.g., the two oldest age classes for scenarios A and C; Appendix A).

## Discussion

Our data support the hypothesis of Waples and Do (2010) that levels of LD in random samples including a number of consecutive cohorts roughly equal to the generation length should provide estimates of per-generation *N*_*e*_. For populations with small *N*_*e*_, the LD method appears to perform relatively well across a variety of sampling strategies. Estimates in our study show moderate bias (typically <20%) and acceptable precision (CV approximately 0.3). For a variety of life histories and sampling schemes, our results show that estimates of per-generation effective size in species with overlapping generations are generally biased downwards, though not severely. This pattern suggests that there was more LD in our datasets than expected, given the sample sizes and effective population sizes simulated in our study. Waples (1991) indicated that additional LD can arise by sampling from age-structured populations because individuals of differing ages are produced by a breeding population that changes gradually over time (i.e., the population is violating the LD method's assumption of random mating because individuals are not the product of a single episode of random mating in the population). As suggested by Waples (1991) and indicated by our simulation results, this bias appears relatively small; however, it is unknown whether this conjecture holds true for demographic and age structure parameters outside the scope of our simulation study. We also found that the sampling method can also introduce additional bias to the estimate. For instance, sampling equal numbers of each age class in the population, or sampling only a small number of age classes, inflated the bias under most of our simulated scenarios (Appendices A and B). It appears that the best estimates of *N*_*e*_ are provided by random samples of reproductively mature individuals.

When the entire population is sampled randomly, the effect of including newborns in the sample is to introduce additional downwards bias. Waples and Yokota (2007) note the same pattern for the temporal method using their barnacle life history. The degree of bias depended not only on the age-specific survival probabilities (as discussed in Waples and Yokota 2007), but also on the reproductive skew in the population and thus on the disparity between *N*_*e*_ and *N*_*b*_. When reproduction increases with age (e.g.*,* C, D, sturgeon, mussel life histories), *N*_*b*_ is reduced relative to *N*_*e*_, and samples including newborns are subject to larger downwards bias. If random samples of adults are difficult to obtain, researchers should seek to include as many age classes as possible in their sample.

The LD method does not perform well when populations with large *N*_*e*_ are considered (Fig. 1). In this case, the method produces some estimates that suggest the population is of very small size, while others indicate that all observed LD could be explained by sample size alone. This result upholds the patterns seen in Waples and Do (2010). Both the temporal (Waples 1989) and LD methods (Waples and Do 2010) work best for small populations, as the genetic signals used to estimate *N*_*e*_ – temporal changes in allele frequencies and LD, respectively – are strongest in these cases. The underlying life history may also influence estimates provided by the LD method. In our study, a life history with extreme reproductive skew, wherein most reproduction is achieved by a very small percentage of the population (scenario D), appears to have led to an upwards bias in the estimates of *N*_*b*_. Furthermore, the patterns of bias among our simulated sampling strategies under this scenario are different from those seen for scenarios A–C. Despite these differences, the LD method still provided useful information about *N*_*e*_ for simulations of the scenario D life history (downwards bias of 15.4% for random samples of adults). Given that our simulation study considered a limited number of life histories, our results should be interpreted with caution.

In general, like most genetic methods for estimating contemporary effective population size (Hare et al. 2011), the LD method is best suited for application in species with small *N*_*e*_. Our simulations show promising results for datasets with *N*_*e*_ as large as 500. Given the average estimates obtained in studies employing the temporal method (Palstra and Ruzzante 2008), the LD method should be appropriate for application in a wide variety of species. Additionally, the LD method has greater expected precision than the temporal method across a broad region of parameter space (Waples and Do 2010). One advantage of the temporal method is that by increasing the time between samples, the signal can be amplified (Waples 1989), allowing reasonable estimates for large populations. However, for species with generation times on the order of years, this will be impractical, particularly in conservation and management situations. When only a single sample is obtainable, the EPA can also estimate effective size in large populations, but requires that a sizeable fraction of the population is sampled to do so (Wang et al. 2010). Previous assessments of the performance of the EPA and LD method suggest that these methods show similar precision (see Waples and Do 2010). The LD method is advantageous in that it does not require detailed information on life-history parameters [required for Jorde-Ryman correction; Jorde and Ryman (1995)] or on the age of individuals [as required for the EPA; Wang et al. (2010)], and it can be applied to single-sample genetic datasets. In situations where *N*_*e*_ is thought to be reasonably small [e.g., species of conservation concern; Palstra and Ruzzante (2008)], even if generations overlap, the LD method should provide useful estimates of *N*_*e*_. As noted by Waples and Do (2010) and illustrated in the sturgeon simulations reported here, a population that is small is unlikely to be mistaken for a large population, whereas a large population may often be mistaken for one of much smaller size.

Our simulated datasets all consist of large samples, both in terms of the number of individuals (*n* = 100) and the number of highly polymorphic loci (20 loci, nine alleles per locus). In some situations, these sample sizes may not be attainable for natural populations, particularly for threatened and endangered species where both effective and census sizes may be quite small. Waples and Do (2010) conducted a thorough investigation of the influence of sample size on estimates from the LD method, finding that increases in the number of individuals, the number of loci, or the number of alleles all improved precision. It is likely that obtaining a sufficient sample, in terms of the number of individuals, the number of alleles, or the number of loci, will be the most significant impediment to applying the LD method in natural systems.

In summary, our results showed that the LD method was broadly applicable to species with iteroparous life histories, although the limited range of life histories considered in our simulation study necessitates caution when the method is applied to estimate *N*_*e*_ in species with overlapping generations. The method provided useful information concerning *N*_*e*_ for species with small effective sizes under a variety of life histories and sampling strategies even when generation time was relatively long. However, in large populations, the method was incapable of providing precise estimates of *N*_*e*_, given our sample sizes. In these cases, the temporal method (if samples can be spaced several generations apart) or the EPA (Wang et al. 2010) may provide more precise estimates. Owing to the rapid ability of the LD method to estimate *N*_*e*_, its application should be particularly fruitful in threatened and endangered species. Waples and Do (2010) noted that studies using the temporal method to estimate *N*_*e*_ could also include a pair of estimates from the LD method. Given that these two approaches provide independent information on *N*_*e*_, their combined use should be encouraged in future applications. Publication of user-friendly software that rapidly calculates the correlation in allele frequencies to provide estimates of *N*_*e*_ (LDne; Waples and Do 2008) should lead to an increase in the number of systems where this method is applied.

## Data archiving statement

The data underlying the main results of this study, including SPIP parameter files, subsampled genotype files, LDne output files, and spreadsheets containing results for all simulated life-history scenarios and sampling strategies, are available at Dryad - doi: 10.5061/dryad.ns40k.

## Appendix A

### Accuracy and precision of estimates for scenarios A–D

Harmonic means of estimated *N*_*e*_, 2.5% and 97.5% estimate quantiles, coefficients of variation (calculated across 1000 replicate datasets), expected CV(*N*_*e*_), relative biases, and the mean numbers of independent allelic comparisons for each of the four primary scenarios simulated in this study and all three allele exclusion criteria (*P*_crit_).

**Table:**

Scenario	Sampling strategy	*P*_crit_	TRUE	Mean estimate	2.5% Quantile	97.5% Quantile	CV(*N*_*e*_)	Exp(CV)	Relative bias	Mean ind. comp.
A	Newborns	0.05	367	351.4	218.6	801.6	6.609	0.211	−0.042	6487.3
		0.02	367	371.6	241.4	761.4	2.303	0.178	0.013	9089.7
		0.01	367	392.4	254.5	728.7	0.319	0.174	0.069	9566.9
	Equal rep.	0.05	378	253.7	166.3	482.1	0.298	0.217	−0.329	6494.2
		0.02	378	268.2	181.8	463.8	0.259	0.183	−0.291	9139.0
		0.01	378	282.9	190.7	489.6	0.257	0.178	−0.252	9611.8
	Random	0.05	378	307.0	190.9	677.0	0.383	0.216	−0.188	6500.3
		0.02	378	324.2	210.5	598.0	0.301	0.183	−0.142	9110.3
		0.01	378	341.7	220.2	619.1	0.294	0.178	−0.096	9573.9
	Adults	0.05	378	304.2	193.9	660.8	0.489	0.216	−0.195	6503.5
		0.02	378	323.7	215.0	612.6	0.302	0.182	−0.144	9161.1
		0.01	378	341.6	230.7	658.1	0.302	0.178	−0.096	9626.9
	3 Youngest	0.05	378	329.8	208.2	690.2	0.375	0.216	−0.127	6527.7
		0.02	378	349.7	228.3	653.4	0.295	0.182	−0.075	9161.5
		0.01	378	367.6	240.8	697.6	0.299	0.178	−0.027	9634.1
	2 Youngest	0.05	378	400.5	238.7	1046.0	0.565	0.216	0.059	6537.5
		0.02	378	428.3	263.5	895.3	0.429	0.182	0.133	9159.3
		0.01	378	453.7	279.5	963.9	0.424	0.178	0.200	9630.6
	2 Oldest	0.05	378	414.8	220.6	1852.5	2.025	0.220	0.097	6292.7
		0.02	378	443.0	245.1	1424.0	0.934	0.185	0.172	8886.9
		0.01	378	464.9	264.2	1462.4	1.421	0.180	0.230	9437.3
B	Newborns	0.05	365	339.6	214.7	730.4	0.370	0.210	−0.070	6461.6
		0.02	365	360.1	234.8	680.7	0.308	0.177	−0.013	9099.6
		0.01	365	379.7	248.6	696.5	0.289	0.173	0.040	9564.4
	Equal rep.	0.05	370.3	249.8	162.4	475.5	0.296	0.213	−0.325	6487.7
		0.02	370.3	265.2	180.9	459.4	0.254	0.179	−0.284	9160.7
		0.01	370.3	279.4	193.0	487.5	0.254	0.174	−0.246	9641.8
	Random	0.05	370.3	298.9	183.1	650.8	0.366	0.213	−0.193	6473.9
		0.02	370.3	315.9	201.8	569.3	0.290	0.179	−0.147	9109.0
		0.01	370.3	333.7	215.0	605.3	0.290	0.175	−0.099	9596.6
	Adults	0.05	370.3	301.9	190.1	601.2	0.342	0.213	−0.185	6493.2
		0.02	370.3	321.8	208.5	566.6	0.271	0.179	−0.131	9151.4
		0.01	370.3	337.5	221.6	601.8	0.270	0.174	−0.089	9635.1
C	Newborns	0.05	273	267.9	178.8	508.2	0.346	0.162	−0.019	6460.3
		0.02	273	282.7	193.9	473.0	0.266	0.137	0.035	9025.1
		0.01	273	299.4	207.7	507.5	0.259	0.133	0.097	9496.8
	Equal rep.	0.05	302.7	231.0	161.0	411.0	0.269	0.177	−0.237	6463.5
		0.02	302.7	243.5	169.6	389.6	0.222	0.150	−0.196	9090.3
		0.01	302.7	258.3	181.7	409.5	0.221	0.146	−0.147	9565.6
	Random	0.05	302.7	258.2	170.8	475.5	0.296	0.177	−0.147	6462.0
		0.02	302.7	273.6	189.1	456.9	0.249	0.150	−0.096	9045.0
		0.01	302.7	289.8	201.8	479.5	0.255	0.146	−0.043	9503.4
	Adults	0.05	302.7	273.9	178.6	526.5	0.307	0.177	−0.095	6477.4
		0.02	302.7	291.2	195.5	519.1	0.261	0.149	−0.038	9096.8
		0.01	302.7	308.1	207.5	557.7	0.270	0.146	0.018	9560.3
	3 Youngest	0.05	302.7	294.2	185.7	557.0	0.309	0.177	−0.028	6471.4
		0.02	302.7	312.4	204.4	535.8	0.260	0.150	0.032	9089.6
		0.01	302.7	329.9	218.7	573.7	0.276	0.146	0.090	9560.7
	2 Youngest	0.05	302.7	305.2	198.4	593.8	0.527	0.177	0.008	6467.1
		0.02	302.7	324.0	214.6	579.4	0.281	0.150	0.070	9080.8
		0.01	302.7	340.5	226.2	610.9	0.282	0.146	0.125	9554.9
	2 Oldest	0.05	302.7	306.0	180.4	796.5	1.038	0.180	0.011	6262.1
		0.02	302.7	328.3	200.0	790.6	0.397	0.152	0.084	8835.8
		0.01	302.7	346.3	210.1	793.2	0.385	0.147	0.144	9374.4
D	Newborns	0.05	85	102.8	70.8	158.3	0.235	0.065	0.210	5992.1
		0.02	85	115.2	81.1	176.9	0.216	0.056	0.355	8160.6
		0.01	85	125.6	89.3	190.1	0.211	0.054	0.477	8580.0
	Equal rep.	0.05	213.8	188.6	131.5	317.8	0.239	0.134	−0.118	6144.7
		0.02	213.8	200.6	138.5	310.7	0.207	0.114	−0.062	8415.6
		0.01	213.8	212.2	149.0	325.5	0.203	0.112	−0.008	8831.0
	Random	0.05	213.8	140.9	94.3	225.8	0.222	0.135	−0.341	6054.6
		0.02	213.8	154.1	105.3	235.1	0.206	0.115	−0.279	8278.5
		0.01	213.8	166.0	114.7	257.3	0.206	0.112	−0.224	8692.9
	Adults	0.05	213.8	167.6	115.4	278.1	0.239	0.134	−0.216	6131.6
		0.02	213.8	180.8	125.1	288.3	0.219	0.115	−0.154	8370.6
		0.01	213.8	193.0	134.8	300.9	0.216	0.112	−0.097	8781.8

## Appendix B

### Accuracy and precision of estimates for small *N*_*e*_ mussel and sturgeon scenarios

Harmonic means of estimated effective sizes, 2.5% and 97.5% estimate quantiles, coefficients of variation (calculated across 1000 replicate datasets), expected CV(*N*_*e*_), relative biases, and the mean numbers of independent allelic comparisons for small *N*_*e*_ sturgeon and mussel scenarios simulated in this study and all three allele exclusion criteria (*P*_crit_).

**Table:**

Scenario	Sampling strategy	*P*_crit_	TRUE	Mean estimate	2.5% Quantile	97.5% Quantile	CV estimate	Exp(CV)	Relative bias	Mean ind. comp.
Sturgeon	Newborns	0.05	207	227.5	155.7	367.9	0.235	0.115	0.0989	7851.4
		0.02	207	251.7	180.9	383.2	0.208	0.095	0.2158	11575.1
		0.01	207	266.2	194.6	409.1	0.204	0.094	0.2862	11863.9
	Equal rep.	0.05	349.3	273.7	183.1	489.6	0.304	0.182	−0.2163	7972.8
		0.02	349.3	299.8	207.5	527.2	0.263	0.150	−0.1417	11689.9
		0.01	349.3	316.5	219.2	541.7	0.267	0.149	−0.0940	11937.8
	Random	0.05	349.3	293.8	190.1	553.8	0.306	0.182	−0.1589	7966.8
		0.02	349.3	321.9	219.1	544.6	0.256	0.150	−0.0784	11653.8
		0.01	349.3	340.1	224.2	562.8	0.251	0.149	−0.0263	11922.2
	Adults	0.05	349.3	315.5	203.7	624.1	0.328	0.182	−0.0967	7966.0
		0.02	349.3	347.4	232.5	644.2	0.276	0.150	−0.0053	11690.0
		0.01	349.3	368.3	246.0	660.2	0.282	0.149	0.0543	11936.9
	5 Youngest	0.05	349.3	272.1	179.9	482.3	0.266	0.183	−0.2211	7886.1
		0.02	349.3	300.9	208.8	489.7	0.240	0.151	−0.1385	11622.0
		0.01	349.3	319.6	223.1	540.1	0.245	0.149	−0.0849	11897.5
	10 Youngest	0.05	349.3	314.4	208.8	606.7	0.311	0.182	−0.0999	7924.4
		0.02	349.3	347.5	235.4	595.4	0.259	0.150	−0.0050	11655.5
		0.01	349.3	368.0	253.9	614.5	0.263	0.149	0.0536	11923.3
	15 Youngest	0.05	349.3	310.0	200.1	584.6	0.298	0.182	−0.1125	7938.6
		0.02	349.3	343.3	237.0	569.2	0.278	0.150	−0.0172	11654.9
		0.01	349.3	362.0	250.2	605.6	0.271	0.149	0.0364	11925.4
	10 Nonconsecutive	0.05	349.3	281.6	185.7	549.6	0.308	0.182	−0.1939	7946.7
		0.02	349.3	311.4	210.1	535.0	0.294	0.150	−0.1086	11662.5
		0.01	349.3	329.2	219.4	568.0	0.303	0.149	−0.0575	11921.6
Mussel	Newborns	0.05	360	369.0	236.5	781.6	0.367	0.186	0.0251	8053.7
		0.02	360	409.6	270.1	746.8	0.285	0.154	0.1378	11779.0
		0.01	360	431.4	285.7	795.7	0.293	0.152	0.1984	11992.3
	Equal rep.	0.05	499.7	372.3	231.0	823.0	0.390	0.251	−0.2550	8117.3
		0.02	499.7	408.8	261.0	802.1	0.325	0.208	−0.1819	11815.1
		0.01	499.7	429.8	276.2	853.8	0.356	0.206	−0.1399	12018.2
	Random	0.05	499.7	375.5	234.2	879.8	0.757	0.251	−0.2485	8125.6
		0.02	499.7	413.0	267.8	859.3	0.364	0.208	−0.1736	11804.5
		0.01	499.7	435.2	278.6	922.8	0.362	0.206	−0.1290	12020.9
	Adults	0.05	499.7	407.3	244.8	942.4	0.537	0.251	−0.1850	8123.4
		0.02	499.7	442.6	279.0	941.6	0.375	0.208	−0.1142	11821.0
		0.01	499.7	464.9	290.0	985.9	0.371	0.206	−0.0696	12021.6
	5 Youngest	0.05	499.7	440.5	261.3	1133.6	0.472	0.252	−0.1185	8080.8
		0.02	499.7	484.7	309.8	1005.0	0.370	0.208	−0.0300	11789.2
		0.01	499.7	512.5	330.5	1134.6	0.381	0.206	0.0256	12004.4
	10 Youngest	0.05	499.7	438.8	266.2	1036.6	0.690	0.251	−0.1220	8112.7
		0.02	499.7	482.7	301.7	1029.8	0.783	0.208	−0.0339	11813.2
		0.01	499.7	509.9	317.5	1134.5	0.474	0.206	0.0203	12015.3
	15 Youngest	0.05	499.7	421.7	250.5	1163.3	1.096	0.252	−0.1561	8084.2
		0.02	499.7	459.8	285.5	977.7	0.507	0.208	−0.0798	11804.1
		0.01	499.7	482.4	304.6	1063.0	0.401	0.206	−0.0347	12014.1
	10 Nonconsecutive	0.05	499.7	368.4	220.1	898.7	0.502	0.251	−0.2627	8099.0
		0.02	499.7	401.6	251.7	868.8	0.561	0.208	−0.1963	11811.1
		0.01	499.7	423.6	266.3	879.0	0.397	0.206	−0.1524	12020.4

## Supporting Information

**Figure S1.** Distribution of *N*_*b*_ (1st panel) and *N*_*e*_ estimates (panels 2–4) for scenario B simulations.

**Figure S2.** Distribution of *N*_*b*_ (1st panel) and *N*_*e*_ estimates 10 (panels 2–4) for scenario D simulations.

**Figure S3.** Distribution of *N*_*b*_ (1st panel) and *N*_*e*_ estimates (panels 2–4) for small *N*_*e*_ mussel simulations.

**Figure S4.** Distribution of *N*_*e*_ estimates 30 for decreasingly random samples from scenario A simulations,

**Figure S5.** Distribution of *N*_*e*_ estimates for decreasingly random samples from scenario C simulations.

**Figure S6.** Distribution of *N*_*e*_ estimates for decreasingly random samples from small *N*_*e*_ mussel simulations.

## References

[b1] Anderson EC, Dunham KK (2005). SPIP 1.0: a program for simulating pedigrees and genetic data I age-structured populations. Molecular Ecology Notes.

[b2] Crandall KA, Posada D, Vasco D (1999). Effective population sizes: missing measures and missing concepts. Animal Conservation.

[b3] Frankham R (2005). Genetics and extinction. Biological Conservation.

[b4] Hare MP, Nunney L, Schwartz MK, Ruzzante DE, Burford M, Waples RS, Ruegg K (2011). Understanding and estimating effective population size for practical application in marine species management. Conservation Biology.

[b5] Hill WG (1972). Effective size of populations with overlapping generations. Theoretical Population Biology.

[b6] Hill WG (1979). A note on effective population size with overlapping generations. Genetics.

[b7] Jorde PE, Ryman N (1995). Temporal allele frequency change and estimation of effective size in populations with overlapping generations. Genetics.

[b8] Laurie-Ahlberg CC, Weir BS (1979). Allozymic variation and linkage disequilibrium in some laboratory populations of *Drosophila melanogaster*. Genetics.

[b9] Miller PS (2008). Preliminary Population Viability Analysis for the Fat Threeridge Mussel (Amblema neislerii).

[b10] Moyer GR, Sweka JA, Peterson DL (2012). Past and present processes influencing genetic diversity and effective population size in a natural population of Atlantic sturgeon. Transactions of the American Fisheries Society.

[b11] Nunney L (1991). The influence of age structure and fecundity on effective population size. Proceedings of the Royal Society of London B: Biological Sciences.

[b12] Nunney L (1993). The influence of mating system and overlapping generations on effective population size. Evolution.

[b13] Palstra FP, Ruzzante DE (2008). Genetic estimates of contemporary effective population size: what can they tell us about the importance of genetic stochasticity for wild population persistence?. Molecular Ecology.

[b14] R Development Core Team (2012). R: A Language and Environment for Statistical Computing.

[b15] Wang J (2005). Estimation of effective population sizes from data on genetic markers. Philosophical Transactions of the Royal Society of London B: Biological Sciences.

[b16] Wang J, Brekke P, Huchard E, Knapp LA, Cowlishaw G (2010). Estimation of parameters of inbreeding and genetic drift in populations with overlapping generations. Evolution.

[b17] Waples RS (1989). A generalized approach for estimating effective population size from temporal changes in allele frequency. Genetics.

[b18] Waples RS (1991). Genetic methods for estimating the effective size of cetacean populations. Reports of the International Whaling Commission.

[b19] Waples RS (2005). Genetic estimates of contemporary effective population size: to what time periods do the estimates apply?. Molecular Ecology.

[b20] Waples RS (2010). Spatial-temporal stratifications in natural populations and how they affect understanding and estimation of effective population size. Molecular Ecology Resources.

[b21] Waples RS, Do C (2008). LDNE: a program for estimating effective population size from data on linkage disequilibrium. Molecular Ecology Resources.

[b22] Waples RS, Do C (2010). Linkage disequilibrium estimates of contemporary Ne using highly variable genetic markers: a largely untapped resource for applied conservation and evolution. Evolutionary Applications.

[b23] Waples RS, Yokota M (2007). Temporal estimates of effective population size in species with overlapping generations. Genetics.

[b24] Waples RS, Do C, Chopelet J (2011). Calculating Ne and Ne/N in age-structured populations: a hybrid Felsenstein-Hill approach. Ecology.

[b25] Wright S (1931). Evolution in Mendelian populations. Genetics.

