# Supplementary material for: Linkage disequilibrium and effective population size when generations overlap
Source: Evol Appl. 2012 Aug 8;6(2):290–302. doi: 10.1111/j.1752-4571.2012.00289.x (PMC3689354; doi:10.1111/j.1752-4571.2012.00289.x)

Supporting Material

Supporting Figure 1: Distribution of N_b (1st panel) and N_e estimates (panels 2-4) for scenario B simulations. This life history matches that simulated for sparrows in Waples and Yokota (2007). Each panel corresponds to estimates from a single sampling strategy (strategies and point values follow Fig. 1).

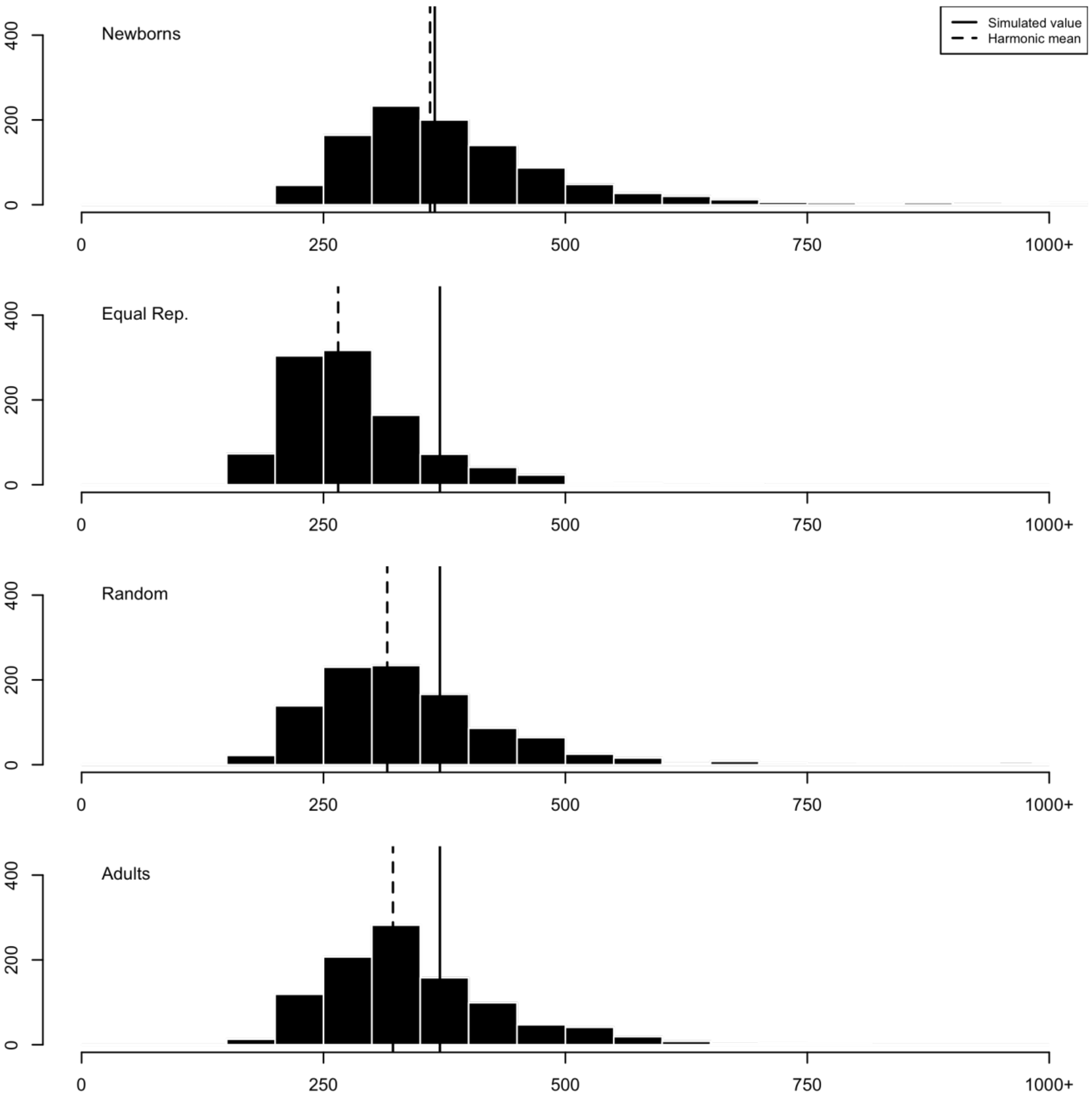

Supporting Figure 2: Distribution of N_b (1st panel) and N_e estimates (panels 2-4) for scenario D simulations. This life history included the maximum reproductive skew simulated, with the oldest age classes contributing the most reproductive effort. Each panel corresponds to estimates from a single sampling strategy (strategies and point values follow Fig. 1).

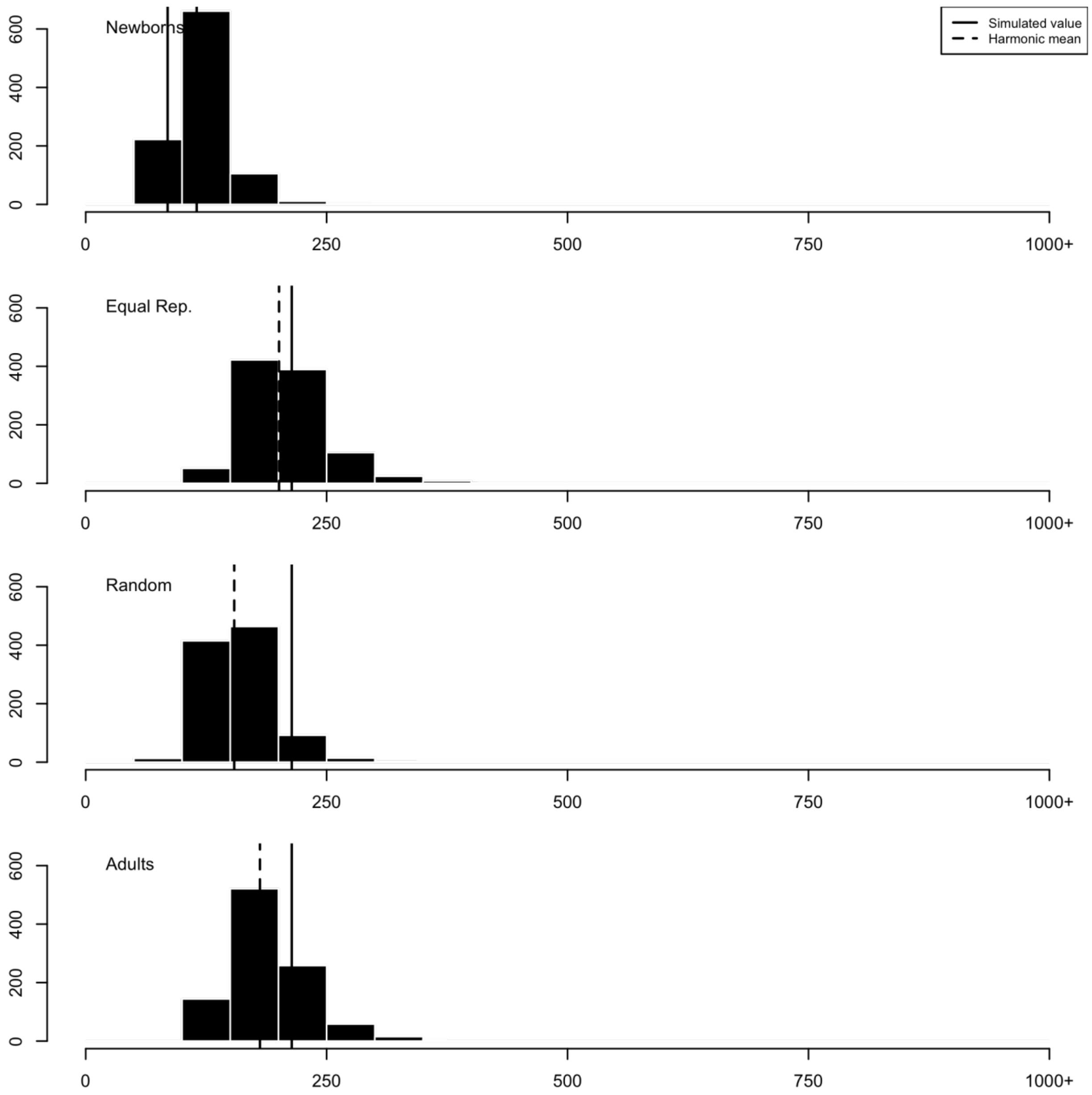

Supporting Figure 3: Distribution of N_b (1st panel) and N_e estimates (panels 2-4) for small N_e mussel simulations. Each panel corresponds to estimates from a single sampling strategy (strategies and point values follow Fig. 1).

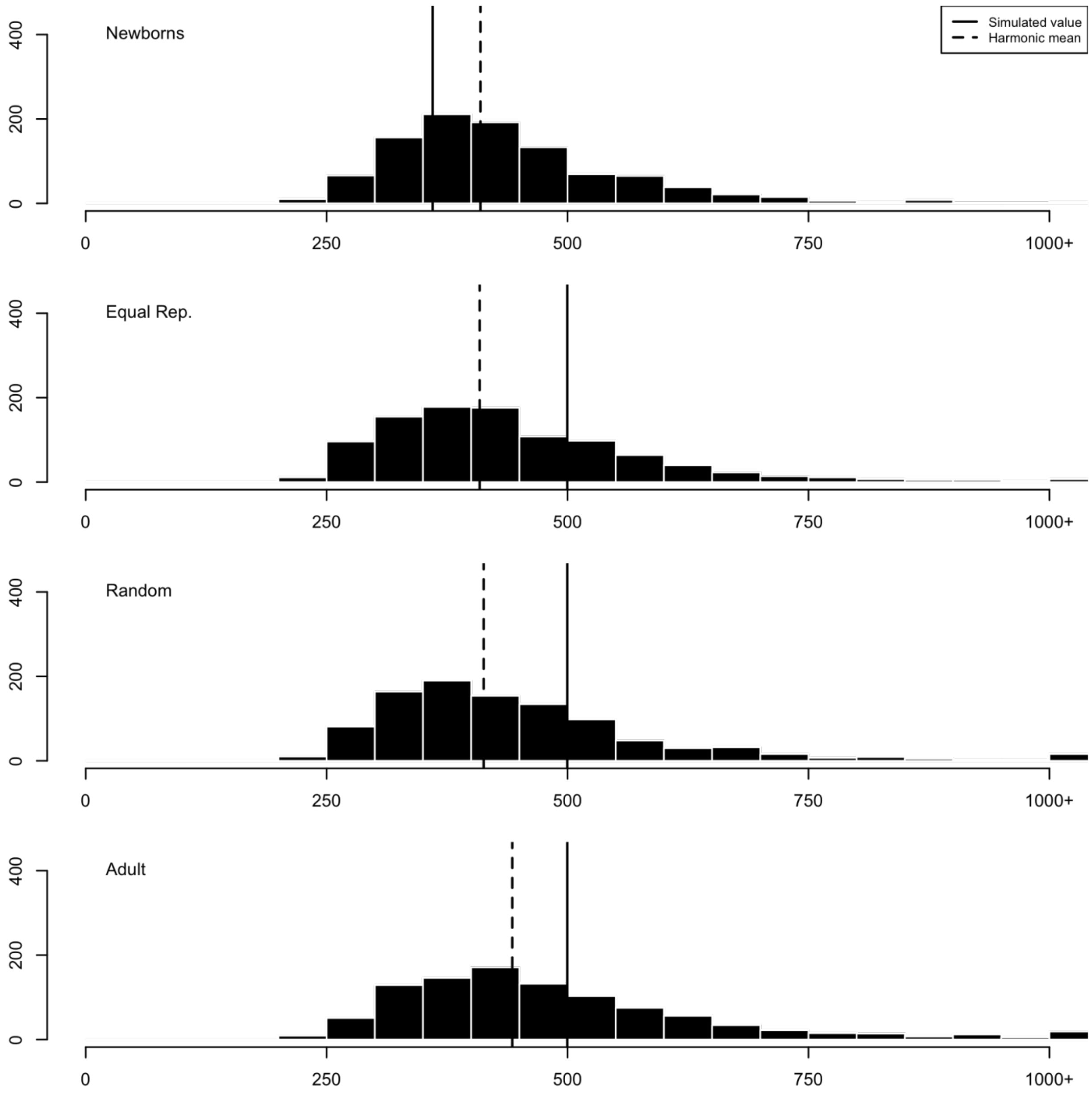

Supporting Figure 4: Distribution of N_e estimates for decreasingly random samples from scenario A simulations. Under this life history, fecundity is constant across age classes. Each panel corresponds to estimates from a single sampling strategy. The number of age classes included in the sample varied among strategies. We include random samples across all age classes for comparison with Fig. 2 (point values given follow Fig. 1).

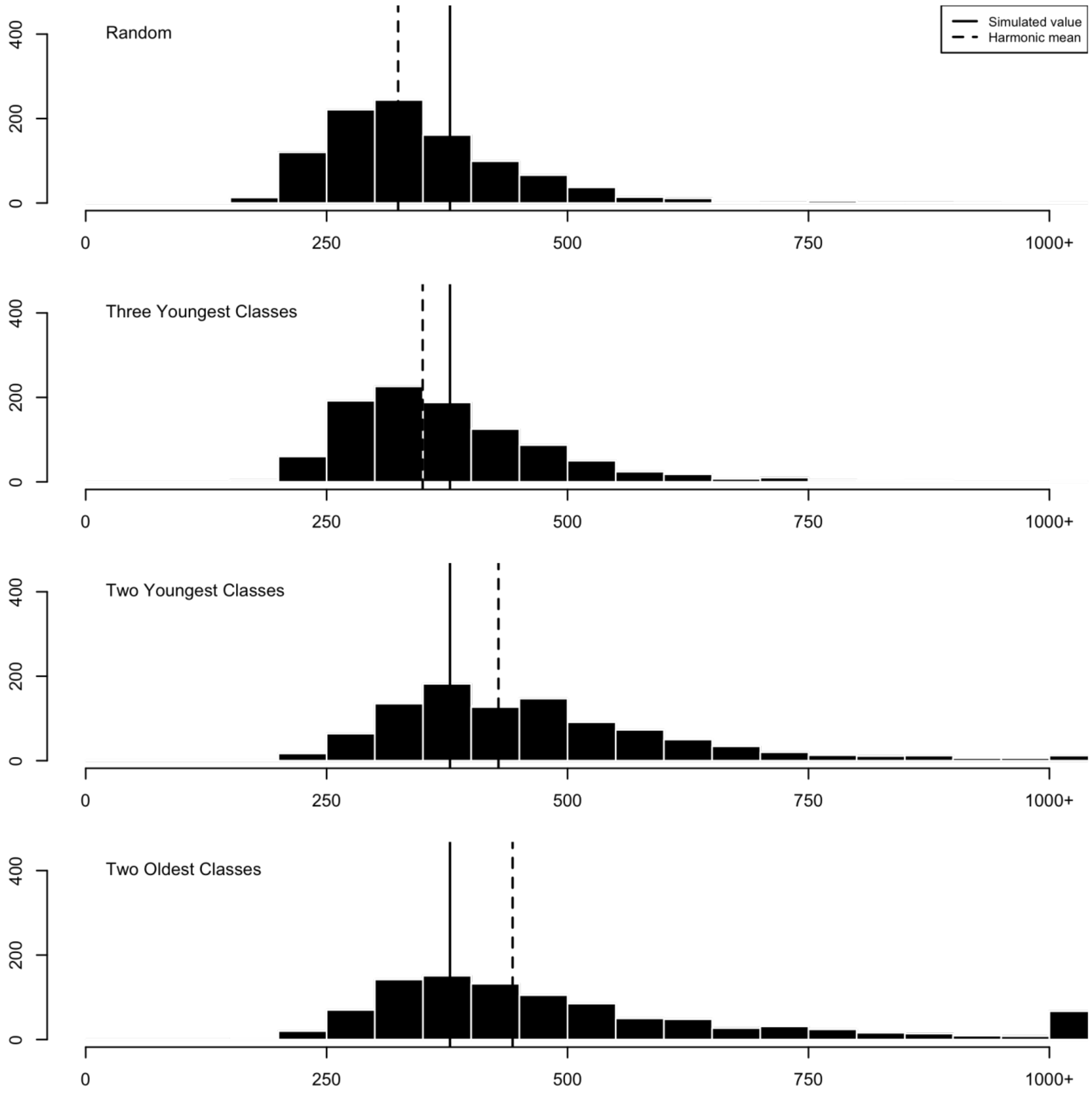

Supporting Figure 5: Distribution of N_e estimates for decreasingly random samples from scenario C simulations. For this life history, fecundity increases linearly with age. Each panel corresponds to estimates from a single sampling strategy. The number of age classes included in the sample varied among strategies. We include random samples across all age classes for comparison with Fig. 3 (point values given follow Fig. 1).

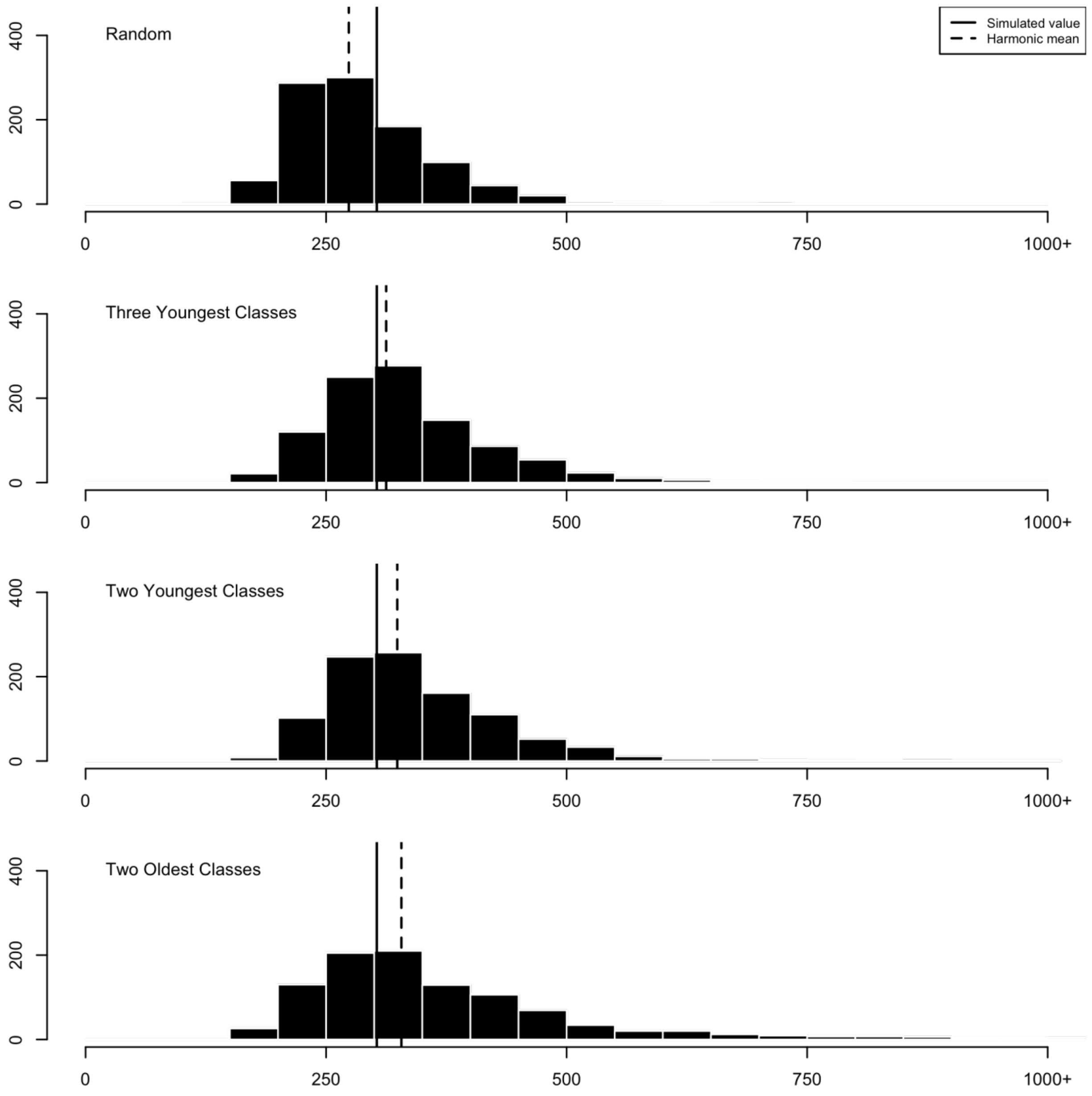

Supporting Figure 6: Distribution of N_e estimates for decreasingly random samples from small N_e mussel simulations. Each panel corresponds to estimates from a single sampling strategy. The number of age classes included in the sample varied among strategies. We include random samples across all age classes for comparison with Supporting Fig. 3 (point values given follow Fig. 1).

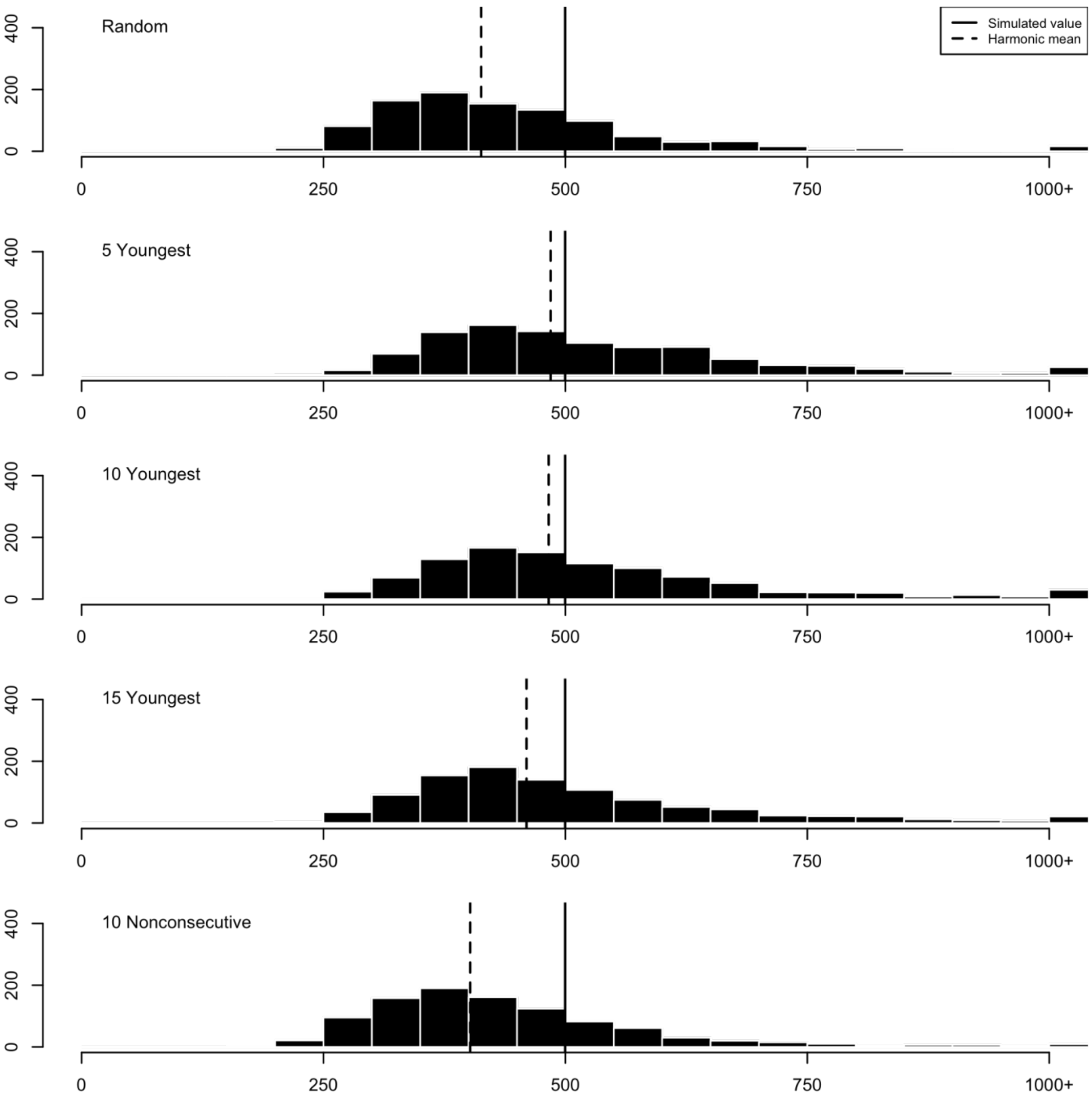

Supplement: Supplementary file 1 [file eva0006-0290-SD1.pdf]
